# A 10-week FIFA 11+ program improves the short-sprint and modified agility T-test performance in elite seven-a-side soccer players

**DOI:** 10.3389/fphys.2023.1236223

**Published:** 2023-11-30

**Authors:** Batool Mohammed Foqha, René Schwesig, Mohamed Amine Ltifi, Thomas Bartels, Souhail Hermassi, Ridha Aouadi

**Affiliations:** ^1^ Higher Institute of Sport and Physical Education of Ksar Said, Tunis, Tunisia; ^2^ Research Laboratory (LR23JS01) “Sport Performance, Health and Society”, Higher Institute of Sport and Physical Education of Ksar Said, University of La Manouba, Tunis, Tunisia; ^3^ Department of Orthopedic and Trauma Surgery, Martin-Luther-University Halle-Wittenberg, Halle, Germany; ^4^ Center of Joint Surgery, MVZ Sports Clinic Halle GmbH, Halle, Germany; ^5^ Physical Education Department, College of Education, Qatar University, Doha, Qatar

**Keywords:** physical performance, warm-up, sprint times, soccer, training program, horizontal jump

## Abstract

**Objective:** The primary objective of this study was to assess the effects of 10 weeks of FIFA 11+ training on the physical performance of elite seven-a-side soccer players.

**Methods:** Twenty-five seven-a-side soccer players were recruited from two senior national teams. The players completed the following protocols during 10 weeks of training: a) FIFA 11+: The FIFA group (*n* = 13) underwent the FIFA 11+ program combined with regular soccer training; b) Dynamic conventional warm-up: The control group (*n* = 12) underwent regular soccer training. Their ability was validated using a pre-test followed by a post-test to measure the sprint performance (5-, 10-, and 20-m sprints), a modified agility T-test (MAT), and a five-jump test (FJT).

**Results:** A comparison of pre- and post-tests for physical performance in each group demonstrated that the FIFA 11+ warm-up significantly improved the 10-m sprinting performance (*p* = 0.034; F = 5.04; η_p_^2^ = 0.17) and reduced the time spent to perform the MAT (*p* = 0.000; F = 23.16; η_p_^2^ = 0.52) in the FIFA group compared with the control group; however, no significant changes were observed in the 5- and 20-m sprints and FJT.

**Conclusion:** The main findings of this research showed that the 10-week FIFA 11+ program led to significant improvements in the 10-m sprint and MAT compared to regular training among elite seven-a-side soccer players. Given these positive outcomes, further studies on the practical implementation and optimization of the FIFA 11+ program are warranted to provide valuable guidance for coaches and athletes, seeking to maximize its benefits in real-world settings.

## 1 Introduction

Soccer is a team sport that encompasses a wide range of physical demands, including short and long sprints, agility, rapid changes in direction, tackling, jumping, and kicking the ball (Haycraft et al., 2017; Seyedi et al., 2023). Consequently, it garners significant attention from researchers and practitioners, aiming to optimize training methods and techniques for enhanced physical performance (Miguel et al., 2021; Torres-Ronda et al., 2022; Ammann et al., 2023). In this context, the development of effective warm-up programs, which prepare soccer players for these challenges, holds paramount importance (Fort-Vanmeerhaeghe et al., 2016).

The FIFA 11+ program has emerged as one such preparatory training program designed specifically for soccer players (Franchina et al., 2023). Endorsed by the International Federation of Football Association (FIFA), it incorporates various cardiovascular and neuromuscular activities aimed at promoting body control, neuromuscular coordination, and postural stability during physical and athletic exercises (Steffen et al., 2013; Franchina et al., 2023). The program comprises 15 exercises grouped into three sections: 1) running exercises with stretching and co-worker contacts; 2) exercises that focus on core and leg strength, agility, and plyometric training; and 3) advanced running exercises and change-of-direction speed (Seyedi et al., 2023).

Compared to other warm-up approaches, the FIFA 11+ program offers several distinct advantages. First, it does not require any additional or specific equipment, making it easily accessible for coaches and players (Bizzini et al., 2013). Additionally, studies have demonstrated that the FIFA 11+ program can elicit acute improvements in performance, including a reduction in 20-m sprint time, an increase in jump height, and improved agility compared to dynamic warm-ups (Bizzini et al., 2013; Pomares-Noguera et al., 2018). Moreover, in addition to its potential benefits for neuromuscular control (Impellizzeri et al., 2013), the FIFA 11+ program also leads to similar improvements in resting oxygen uptake, core temperature, and lactate levels when used as conventional warm-ups, indicating its effectiveness in preparing players for the physical demands of the game (Pomares-Noguera et al., 2018).

An essential benefit of the FIFA 11+ program lies in its established effectiveness in mitigating the incidence of sports-related injuries, notably those affecting the lower extremities, including knee injuries (Owoeye et al., 2014; Silvers-Granelli et al., 2015; Al Attar et al., 2021; Miguel et al., 2021). This reduction in injury incidence is attributed to improvements in the neuromuscular control of the trunk and lower limb achieved through the regular implementation of the FIFA 11+ program (Daneshjoo et al., 2012). It has been suggested that the high neural demand of plyometric training (e.g., countermovement jump for maximum height and double-leg hops in different directions) included in FIFA 11+ can provide a stimulus that aligns with proliferation in neural coordination and central nervous system maturation, notably observed during pre-pubescence (Lloyd et al., 2016). Additionally, athletes who participate in FIFA 11+ benefit not only from neurophysiological adaptations but also from dynamic correspondence, specifically in terms of horizontal force production vectors, which contribute to improved performance (Arede et al., 2022).

However, no studies have investigated the effectiveness of the “FIFA 11+” warm-up. It is well known that a sufficient warm-up is able to improve the performance and reduce the injury risk (Bizzini et al., 2013). With this background and in detail, we consider the following main targeted physiological factors: an increase in muscle and core temperature, anaerobic metabolism, nerve conduction rate, blood flow, and oxygen delivery to muscles (Bishop, 2003). The dynamic exercises that are covered by “FIFA 11+” are potentially enough to induce physiological modifications suitable for an appropriate warm-up. This warm-up protocol has been the subject of several studies in numerous age groups (Neto et al., 2017). Some encouraging findings from a randomized controlled trial suggested that the FIFA 11+ program may be helpful in producing a significant improvement in vertical jump and change-of-direction speed among 9–11-year-old female soccer players (Parsons et al., 2019). However, despite the number of studies exploring the chronic effects of FIFA 11+ on some measures of physical performance (Daneshjoo et al., 2012; Impellizzeri et al., 2013; Steffen et al., 2013), postural stability, sprinting, and jumping ability (Daneshjoo et al., 2013; Impellizzeri et al., 2013), the findings of the literature are conflicting and hence not conclusive (Robles-Palazón et al., 2016). In addition, its short- or long-term effects on physical performance, especially in elite seven-a-side soccer players, remain relatively unexplored.

Therefore, the first aim of this present study was to evaluate the effects of 10-week FIFA 11+ on several physical performance measures (sprinting, jumping, and change-of-direction speed) in elite seven-a-side soccer players, in order to find out whether this program can be considered an appropriate warm-up routine for soccer players. The second aim was to compare the effects of the 10-week FIFA 11+ warm-up in an experimental group and a regular warm-up in a control group on short sprint, change of direction, and horizontal jump performances in elite senior seven-a-side soccer players. Due to the lack of comparable reports, we propose the null hypothesis that there will be no difference in the physical performance tests between FIFA 11+ and regular warm-up programs.

## 2 Materials and methods

### 2.1 Sample of subjects

In this study, we conducted *a priori* power calculations using G*power software to determine the necessary sample size. The purpose of these calculations was to ensure that our study has a high probability (80%) of detecting a significant effect, given a 5% level of significance (alpha). In other words, we aimed to have a strong likelihood of identifying meaningful results if they exist in our data. The estimated effect size of 0.50 represents the magnitude of the difference we expect to observe between conditions. This value is crucial as it helps us gauge the practical significance of our findings. By performing these power calculations, we aimed to optimize the study’s design and sample size, ensuring that our research can provide meaningful and statistically sound conclusions. Based on G*Power, 12 participants per group for a total of 24 participants were needed. Participants included 25 soccer male athletes (FIFA group, *n* = 13; control group, *n* = 12) from two teams. The two teams were randomly allocated to the FIFA group and control group using the simple random sampling technique between the two teams using a coin flip (Melnyk and Morrison-Beedy, 2020). A unique identifier was assigned to each team (the two teams were labeled as the control group and FIFA group). In order to ensure the confidentiality of the randomization sequence, this process was conducted by another independent assessor researcher external to the study.

Based on the players’ baseline characteristics including anthropometric data and the history of previous injuries, which were given by the medical staff (team physician and physiotherapist) for each individual player, none of the players suffered from any musculoskeletal disorders, and they were required not have been dependent on a medical procedure on the lower limb during 3 months prior to the study. The study involved participants from the first national championship soccer (Division-I), and it is important to clarify that the participants were seven-a-side professional soccer players. They underwent training sessions for 4–5 days a week (∼90 min per session) and played one match over the weekend. All participants possessed more than 8 years of experience in soccer training and competition and took part in national championship at the time of investigation. The age and anthropometric characteristics (height and body mass) of the groups are listed in Table 1.

**TABLE 1:** Characteristics of both groups (mean ± SEM, *n* = 25).

Group	Age (years)	Height (cm)	Body mass (kg)
Control	25.6 ± 1.25	179 ± 1.35	71.9 ± 2.07
FIFA	28.1 ± 0.79	178 ± 1.21	77.9 ± 2.30

After randomization of the groups and before beginning the FIFA 11+ program, two soccer players were excluded because of leg injury.

**FIGURE 1:**
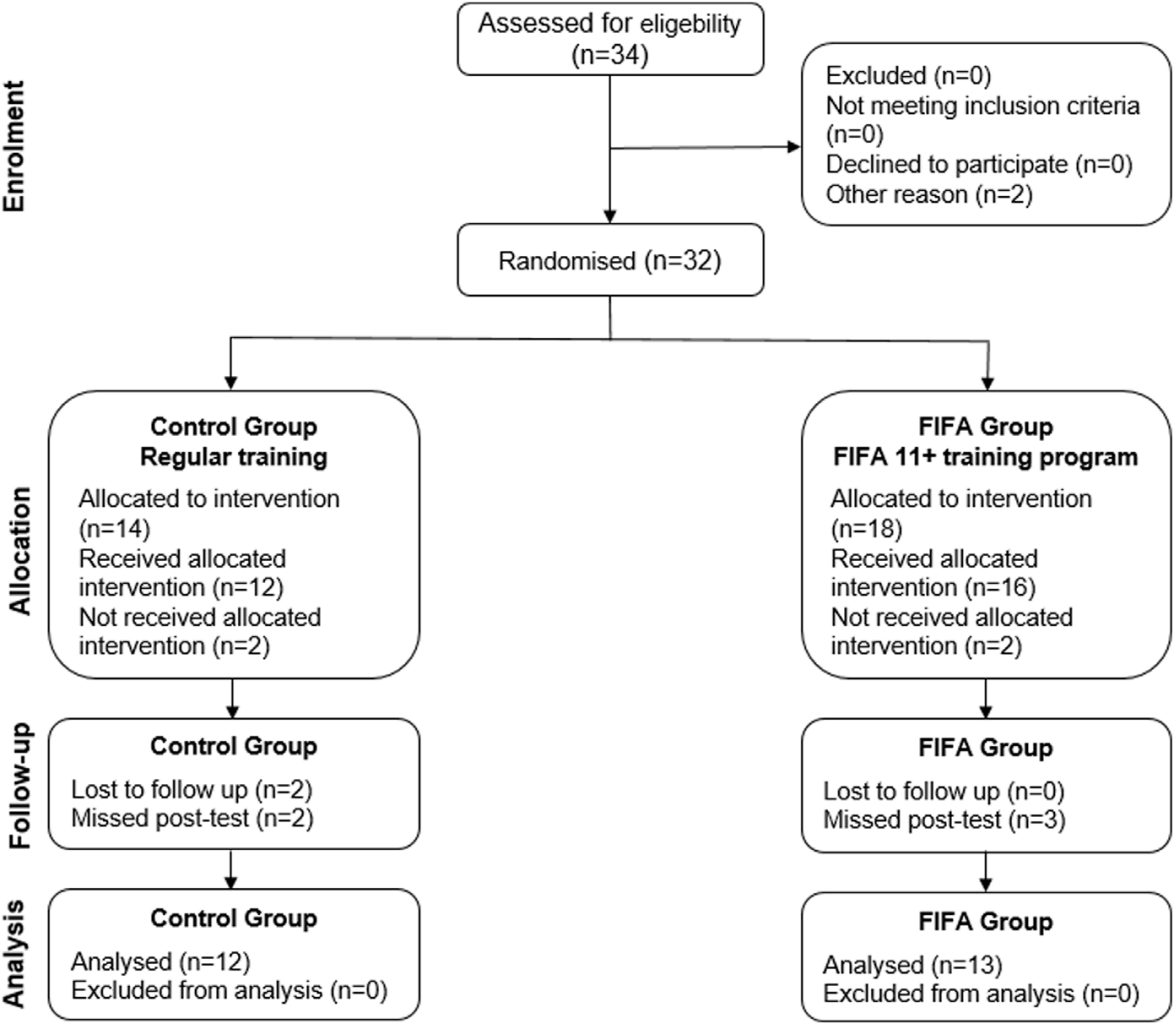
Consort diagram includes detailed information about the interventions received and the number of participants through each stage.

The study adopts a pre-test–post-test control group character design with an intervention period of 12 weeks. Two teams were used (Figure 1): the FIFA group (*n* = 18), which is considered the experimental group, and the control group (CG; *n* = 14).

After randomization of the groups and during the intervention, five soccer players from the FIFA group and two players from the control group were injured. The FIFA group was then reduced to thirteen players, while the CG was reduced to twelve players.

### 2.2 Experimental design

Two weeks before the pre-test measurements, all subjects completed two familiarization trials of all procedures except the anthropometric characteristics. Subjects of the two groups (control and FIFA groups) performed measurements of speed (i.e., 5-, 10-, and 20-m sprints), modified agility T-test (MAT), and five-jump test (FJT) before and after training.

Pre- and post-tests were conducted over 2 days with an interval of 72 h between each session. The test measurements were completed using the same testing order for all performance tests on the same soccer field. The details of the study were explained to the participants by the researchers. All procedures were approved by the Manouba University Institutional Review Committee (Tunisia) for the ethical use of human participants and were conducted in accordance with the Declaration of Helsinki. The participants were informed that they were free to withdraw from the study at any time without penalty. Individual written consent was obtained from all participants after they had received both oral and written explanations of the experimental procedure and its possible risks and benefits.

The study was realized over a period of 12 weeks during January, February, and March as part of an official competitive season. To minimize the effects of circadian rhythms on performance, all sessions were conducted at the same time of the subjects’ regular training session time (in the evenings) and at same temperature and humidity ranges (22°C–24°C and 38%–42%, respectively). After the completion of the pre-test, the control group participants were asked to continue their regular training (which consists of standard jogging, ball exercises, and whole-body stretching). In addition to regular training, the FIFA group performed the FIFA 11+ program three times a week, with a mean of 20 min per session. Three training sessions per week (∼90 min per session) on different days (non-consecutive days) were performed. In addition, the participants played an official match per week.

**FIGURE 2:**
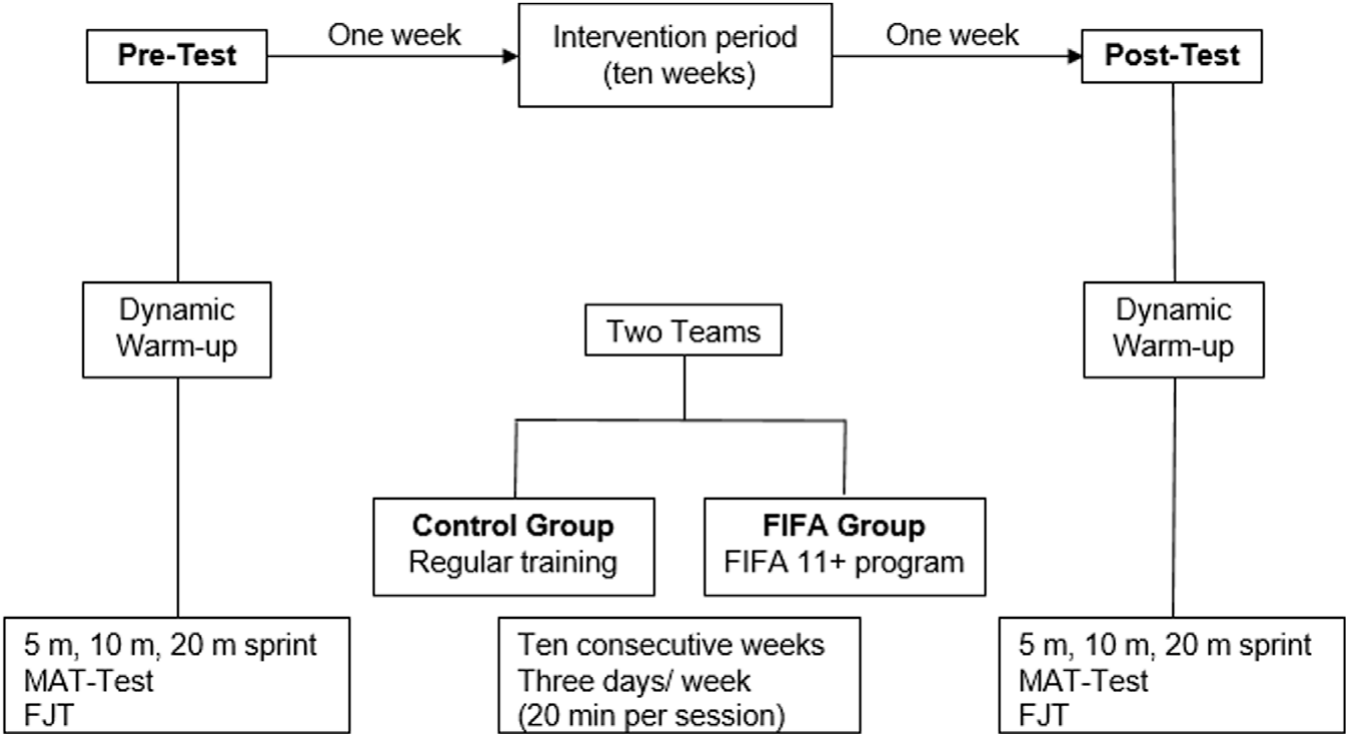
Schematic representation of the study design. R, randomization; FJT, five-jump test; MAT, modified agility T-test.

The data collection involved a baseline phase followed by 10 weeks of the FIFA 11+ program and a final phase at the end of the FIFA 11+ program (Figure 2).

### 2.3 Testing procedures

#### 2.3.1 Modified agility T-test (MAT)

**FIGURE 3:**
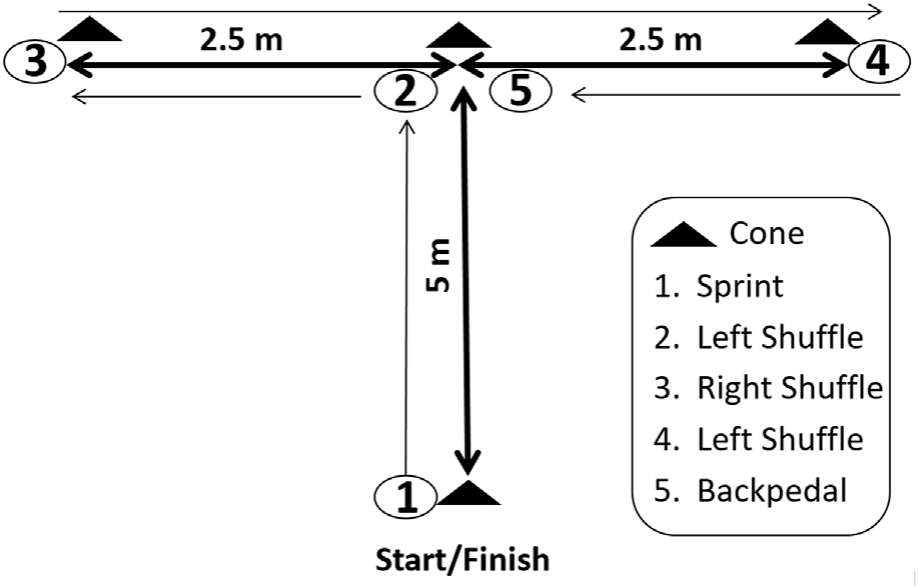
Modified agility T-test (MAT) showing the movement patterns which should be performed.

The MAT was determined to examine the change-of-direction speed during forward sprinting, lateral leftward and lateral rightward shuffling, and back-pedaling (Sassi et al., 2009). A photocell system (Microgate, Bolzano, Italy) was used to measure the time. Each player performed two trials with at least 2-min rest between trials, and the best MAT time was used for the analysis. Subjects were instructed to begin the test with both feet placed on a marked line of 30 cm behind the starting gate (Mathisen and Danielsen, 2014) and 1) run as quickly as possible forward linear (5 m) to cone 2 and then touch its base with their right hand (Figure 3). 2) Next, facing forward and without crossing feet, they should sidestep to the left 2.5 m to cone 3 and touch its base with the left hand. Afterward, they must 3) sidestep to the right 5 m to cone 4, and touch its base with the right hand. Then, they should sidestep back 2.5 m to cone 2, touching its base and run back as quickly as possible toward the finish line (Figure 3; Sassi et al., 2009).

#### 2.3.2 Sprint test

The 20-m sprint test was preceded by a standardized warm-up with two sub-maximal 20-m sprints. Four paired photocells (Microgate, Bolzano, Italy) were used and positioned in a straight line at distances of 0, 5, 10, and 20 m along the course. The paired photocells, separated by a distance of 1.5 m (Yeadon et al., 1999), were located 1 m above the ground at the starting and finishing lines. The first sprint was started 30 cm behind the starting photocell gate, and time measurement started when the subject traversed the first gate. Players performed two maximal 20-m sprints, and the best performance was used for the statistical analysis. Times over 5, 10, and 20 m were recorded. A 5–8-min rest interval of recovery was allowed between the two trials, and the fastest time for each distance was retained to be analyzed.

#### 2.3.3 Five-jump test (FJT)

This test was used to evaluate player’s lower limb explosive power. For the FJT, each player should perform, from an upright standing position, five forward jumps by alternating left- and right-leg ground contacts and tried to cover a maximal distance. At the start of the FJT, each player with joined feet had the choice to select which foot to put first. Distances covered when performing the five-jump test were measured using a tape measure (Ayed et al., 2020).

### 2.4 Training program

The weekly soccer training regimen was followed by both teams (three 90-min sessions per week and one match per week). The study assistants paid both teams a visit at least once per week to verify whether the groups did the suggested soccer training with a regular warm-up. The same weekly soccer training regimen was followed by both teams with three 90-min sessions per week and one match per week. The training including FIFA 11+ was conducted by the habitual coach of the FIFA group (i.e., the same coach of the team) after identifying and presenting the FIFA 11+ program and completing two familiarization trials of all procedures. The FIFA 11+ warm-up comprised a program with a total duration of 20 min and included three levels of difficulty, which depends on the athletes’ age and their physical aptitude. Therefore, level II of difficulty, which was completed during the familiarization sessions, was chosen for this study. It consists of three parts (Impellizzeri et al., 2013). The first part consists of running exercises; the second part focuses on core and leg strength, balance, and plyometric/agility; and the third part includes running exercise combined with direction changes (cutting movements). In this research, during the experimental period, the FIFA group followed the third and the hardest level, while the control group performed their usual soccer training, as applied by Trajković et al. (2020) (Table 2).

**TABLE 2:** ‘‘FIFA 11+’’: Exercises, duration, and intensities of the structured warm-up program used (F-MARC).

Exercise	Duration (min)
Part 1: Running	8
Straight ahead, hip out, hip in, circling partner, shoulder contact, and quick forward and backward (six running items, each item performed in two sets)	
Part 2: Strength and plyometric and postural stability	10
The bench	
Static, alternate legs, and one leg lift and hold (three items, each item performed in three sets)	
Sideways bench	
Static, raise, and lower hip, with leg lift (three items, performed in three sets on each sides)	
Hamstring	
Beginner (3–5 repetitions, one set), intermediate (7–10 repetitions, one set), and advanced (12–15 repetitions, one set) (three items)	
Single-leg stance	
Hold the ball, throwing the ball to the partner, and test your partner (three items, each item performed in two sets)	
Squats	
With toe raise, walking lunges, and one-leg squats (three items, each item performed in two sets)	
Jumping	
Vertical jumps, lateral jumps, and box jumps (three items, each item performed in two sets)	
Part 3: Running exercise	2
Across the pitch, bounding, plant, and cut (three items, each item performed in two sets)	

### 2.5 Data analysis

Data are presented as the mean ± standard error of the mean (SEM). SPSS version 28.0 for Windows (IBM, Armonk, NY, United States) was used for all statistical analyses. Normality distribution was determined using the Shapiro–Wilk test (*p* < 0.05). Moreover, the sphericity and homogeneity were checked using Mauchly’s and Levene’s tests, respectively. The effects of the interventions on the physical parameters were analyzed using a 2 × 2 (control and FIFA groups x pre- and post-test) two-way repeated measures analysis of variance (ANOVA). Test–retest reliability was established using the intraclass correlation coefficient (ICC). The absolute reliability, measured by the coefficient of variation (CV%), was calculated by dividing the SEM and the sum of the average attempts and multiplied by 100 (Macadam et al., 2017). The interpretation of the ICC values was based on Shrout and Fleiss (1979). The ICC was considered to show excellent relative reliability (inter-subject variability) when >0.75, fair to good when 0.40–0.75, and poor when <0.40. The CV can be defined as good with values <10% (Brughelli and Van Leemputte, 2013). The statistical significance was set at *p* < 0.05. Effect size, presented by partial eta-squared (η_p_^2^), was determined to assess the magnitude of the effects (Macadam et al., 2017). Partial eta-squared (η_p_^2^) was chosen because it is particularly suitable for assessing the proportion of variance explained by each factor while accounting for the interactions among factors and the impact of covariates. The more independent the variables included in the model, the less accurate the use of the eta-squared compared to the partial eta-squared. The use of this effect size aligns with the classification provided by Cohen (1988): 0.01 < η_p_^2^ < 0.06 (small effect), 0.06 < η_p_^2^ < 0.14 (medium effect), and η_p_^2^ ***≥*** 0.14 (large effect).

## 3 Results

At baseline, there were no significant differences in the anthropometric characteristics and physical performance parameters between the groups (*p* ≥ 0.05). Mean repeated measure indices displayed strong inter-trial (ICC = 0.86–0.94; CV%: 4.5–7.0) and intersession reliability (ICC = 0.89–0.95; CV%: 2–14). A coefficient of variation (CV%) ranging between 4.5% and 7% is generally considered quite favorable when assessing reliability. The goal of having a CV of less than 10% to denote reliability is widely accepted within the scientific community (Turner et al., 2015), which suggests that our study exhibits relatively low variability in relation to the mean. This means that the measurements are relatively consistent and precise. The differences between pre- and post-tests in the FIFA 11+ warm-up were determined for different performance variables in each group. The results demonstrated that the FIFA 11+ warm-up significantly improves the 10-m sprinting performance in the FIFA group. In fact, the time performance was significantly reduced from 1.95 ± 0.05 s to 1.88 ± 0.08 s for the 10-m sprint (*p* = 0.034; F = 5.04; η_p_^2^ = 0.17). However, no significant changes were observed for the 5-m (*p* = 0.531; F = 0.68; η_p_^2^ = 0.03) and 20-m sprints (*p* = 0.051; F = 4.23; η_p_^2^ = 0.15).

Furthermore, the time spent to perform the MAT was significantly reduced in the FIFA group from 6.43 ± 0.47 s to 5.51 ± 0.27 s (*p* < 0.001; F = 23.16; η_p_^2^ = 0.52) in the post-test compared to that observed in the pre-test (Table 3). The differences observed in the pre- and post-tests for the remaining studied physical performance parameters for the FIFA 11+ program (5- and 20-m sprints and FJT) were not significant. However, no significant effect was observed between the values of pre- and post-tests in the control group who underwent the habitual training program (Table 3).

**TABLE 3:** Descriptive statistics (mean ± SEM) for sprint (5, 10, 20 m), modified agility T-test (MAT), and five-jump test (FJT) performance. Relevant mean differences (η_p_^2^ ≥ 0.14) marked in bold.

Test	Control group mean ± SEM (s)		FIFA group mean ± SEM (s)		Group			Time			time * group		
	Pre-test	Post-test	Pre-test	Post-test	F	p	η_p_^2^	F	p	η_p_^2^	F	p	η_p_^2^
5-m sprint	1.08 ± 0.02	1.09 ± 0.01	1.14 ± 0.02	1.10 ± 0.02	0.40	0.53	0.02	0.68	0.42	0.03	0.09	0.77	0.01
10-m sprint	1.81 ± 0.03	1.87 ± 0.02	1.95 ± 0.18	1.88 ± 0.06	5.04	0.03	0.17	4.02	0.06	0.14	1.40	0.25	0.05
20-m sprint	3.13 ± 0.03	3.18 ± 0.03	3.22 ± 0.04	3.24 ± 0.03	4.23	0.05	0.15	3.21	0.09	0.12	0.01	0.92	0.00
MAT	6.57 ± 0.07	6.22 ± 0.13	6.43 ± 0.13	5.51 ± 0.08	23.2	<0.001	0.52	20.1	<0.001	0.49	1.01	0.33	0.05
FJT	11.6 ± 0.10	11.6 ± 0.12	11.3 ± 0.20	11.5 ± 0.22	0.89	0.46	0.02	0.88	0.36	0.03	0.45	0.51	0.02

SEM: standard error of measurement; η_p_^2^: partial eta-squared.

Time: This is an independent variable, and it represents two different time points—pre-test and post-test. These time points were used to measure changes over time in the study.

## 4 Discussion

The aim of this study was to investigate the effects of the FIFA 11+ warm-up program over a 10-week period on the physical performance of elite seven-a-side soccer players. The results of our study indicated that after completing the program for 10 weeks, certain physical performance parameters, such as the 10-m sprints and the MAT, showed significant improvements as a result of the training stimuli provided by the FIFA 11+ program.

The observed effects of the FIFA 11+ program on physical performance were generally in the range of moderate to substantial, and they align with findings commonly reported in the existing literature. Numerous studies have reported improvements in various physical performance variables in soccer players following the implementation of the FIFA 11+ program. These improvements encompass a wide range of aspects. In particular, Daneshjoo et al. (2013) and Kilding et al. (2008) reported benefits in jumping height, 20-m sprint time, and Illinois agility tests compared to traditional warm-up routines. These improvements were observed in young male professional football players aged between 17 and 20 years (Daneshjoo et al., 2013) and preadolescent football players with an average age of 10.4 ± 1.4 years (Kilding et al., 2008). Both groups experienced these positive effects after participating in the FIFA 11+ program three times per week for 2 months.

Similarly, Ayala et al. (2017) noted significant improvements in sprinting speed, both in 10- and 20-m sprints, among young male amateur soccer players with an average age of 16.8 ± 0.7 years. These improvements were observed after the participants engaged in the FIFA 11+ program for 4 weeks, with three sessions per week. Furthermore, Arede et al. (2022) reported a significant decrease in 0–20-m sprint times and change-of-direction times in young male soccer players with an average age of 11.2 ± 0.7 years. These findings collectively suggest the positive impact of the FIFA 11+ program on various aspects of physical performance in different age groups of soccer players.

For a field sport athlete, the distance from 5 to 10 m in a sprint could be viewed as a transitionary period between initial acceleration and peak velocity. Training protocols that encourage high force production (i.e., weights or plyometric training) may be required to enhance performance in the transition from acceleration to maximum velocity in field sports (Lockie et al., 2012). Regarding our results, the significant improvement in the 10-m sprint performance after the FIFA 11+ program, without a corresponding significant improvement in the 5-m sprint, can be explained by the unique demands of these two sprint distances and the specific nature of the training protocols involved. In a sprint, the first 5 m typically involves initial acceleration (Lockie et al., 2012), where athletes need to generate quick bursts of speed from a stationary position. This phase relies heavily on explosive power and rapid acceleration, and it can be influenced by factors such as the strength of the leg muscles, coordination, and technique. It is during this phase that a high ground reaction force is critical. Therefore, it is possible that the FIFA 11+ program, while beneficial for the transition phase and peak velocity, may not specifically target the requirements of the 5-m sprint phase, which demands rapid acceleration. This could explain the absence of a significant improvement in the 5-m sprint, despite the improvements observed in the 10-m sprint.

The FIFA 11+ program, while effective in enhancing aspects of performance, may primarily focus on elements that benefit the transition from acceleration to maximum velocity, which often occurs in the 10-m phase of a sprint. This transition phase is different from the initial acceleration phase and places different demands on the athlete. It involves maintaining and increasing the speed, and it may benefit from the development of maximal strength, power, and neuromuscular coordination.

It seems that soccer players who participated in FIFA 11+ have demonstrated neurophysiological adaptations, as indicated by Arede et al. (2022). This study supports previous research suggesting that plyometric training is highly effective in improving short-to-medium sprinting times (under 20 m). Following plyometric training, sprint training emerges as the most effective method for further improving sprint times in participants at the pre-peak height velocity stage (Rumpf et al., 2012). Notably, the FIFA 11+ protocol incorporates sprinting-based activities, including 40-m sprints at 75%–80% of maximum speed, involving movements that utilize the stretch-shortening cycle. These activities have the potential to enhance an individual’s rate of force development, impulse generation, and muscle stiffness—neurophysiological adaptations associated with optimized sprint performance (Haff and Triplett, 2016).

However, the lack of improvement in the FJT could be due to various factors, including the duration of the training program, the specific demands of these tests, and the individual responses of the athletes. It is possible that more extended or targeted training may be needed to obtain significant changes in longer sprints and explosive power measured by the FJT.

Additionally, the program has been associated with improvements in dynamic balance (Steffen et al., 2013), jump height (Blazevich and Babault, 2009), and muscle strength and power (Steffen et al., 2013). In direct comparisons to a standard dynamic warm-up, researchers have consistently observed enhancements in agility times and improved endurance of trunk muscles among participants who followed the FIFA 11+ program (Trajković et al., 2020). These findings collectively emphasize that the FIFA 11+ warm-up program effectively enhances various aspects of physical performance among soccer players.

However, in contrast to our findings, some studies have reported no improvements in several specific physical performance aspects relevant to soccer practice. Notably, our results regarding sprint tests contradicted the outcomes of previous studies conducted on male amateur soccer players aged 23.7 ± 3.7 years, specifically in the context of sprints (10 and 20 m) and the agility T-test (Impellizzeri et al., 2013). One potential explanation for these contradictory results could be the differing durations of the intervention phases within the training regimens used by the respective authors. For instance, Impellizzeri et al. (2013) found no significant improvement in sprint performance among professional soccer players following a 2-month FIFA 11+ training program that incorporated soccer-specific content. Similarly, after a 9-week period of implementing the FIFA 11+ program, Impellizzeri et al. (2013) reported comparable results among amateur soccer players. These variations in outcomes may be attributed to differences in the duration of the intervention programs and the specific content of the training, highlighting the importance of considering these factors when evaluating the impact of warm-up programs on physical performance in soccer players.

To assess this, the players substituted their regular warm-up routine with “FIFA 11+” three times a week for a total of 9 weeks. During the first 3 weeks, they followed level 1 of the program. In the following 3 weeks (weeks 4 to 6), they advanced to level 2, and in the last 3 weeks (weeks 7 to 9), they progressed to level 3. The post-test results of agility performance metrics in our study were consistent with the findings from other research studies (Zarei et al., 2018; Trajković et al., 2020). However, it is worth noting that some authors have reported that the Illinois agility test results remained unaffected by FIFA 11+ exercises, as demonstrated by Impellizzeri et al. (2013) and Lopes et al. (2019). It is important to recognize that while there is a relationship between the Illinois agility test and the modified T-test, there are key distinctions between the two tests. The MAT is not a continuous running test; instead, it involves multiple stopping and re-activation phases. This fundamental difference sets the T-test apart from the Illinois tests, as highlighted by Muniroglu and Subak, (2018).

An important argument to consider is that exercise-based injury prevention programs have the potential to improve relevant performance measures, as demonstrated by Maffulli et al. (2011). Existing evidence suggests that injury prevention programs can have beneficial effects on various performance parameters among soccer players. These effects encompass improvements in anaerobic power (Kilding et al., 2008; Daneshjoo et al., 2013), sprint performance (Kilding et al., 2008), neuromuscular control (Impellizzeri et al., 2013), and agility performance (Daneshjoo et al., 2013; Rössler et al., 2016).

The FIFA 11+ program has been shown to have potential benefits for neuromuscular control; however, Impellizzeri et al. (2013) noted that its impact on the overall athletic performance of soccer players may be limited. Notably, in their study, even though players undergoing the FIFA 11+ program tended to show improvements in the Illinois agility test and sprint performance only after 9 weeks, these improvements did not reach statistical significance (p for interaction 0.126 and 0.042, respectively). The absence of significant improvements in these performance measures could be attributed to the training stimulus provided by the FIFA 11+ program, which may have been insufficient in terms of both intensity and duration to elicit a notable response in the measured soccer performance parameters. Impellizzeri et al. (2013) suggested that while the amount of plyometric and agility training in the FIFA 11+ program might be adequate for enhancing neuromuscular control, it may fall short of the intensity typically employed to improve these aspects in soccer players, particularly through dedicated plyometric training. It is important to note that direct comparisons of the results of these studies with those of our current study should be done cautiously due to differences in study populations and the presence of various uncontrolled confounders, both experimentally and statistically, which may have influenced the outcomes.

Steffen et al. (2013) indicated that the lack of significant improvement in physical performance following the FIFA 11+ program could be attributed to several factors. One key factor is the relatively low intensity of the FIFA 11+ program itself compared to the high-intensity demands of soccer practice. In our study, we specifically used the second level of the FIFA 11+ program, which is designed to be more intensive than the first level but less intensive than the third level. While the second level represents an intermediate intensity option within the program, it is essential to note that the FIFA 11+ program offers a progression through three distinct levels to cater to various fitness levels and demands. This allows for flexibility in tailoring the warm-up to suit the needs of different groups of soccer players. The primary objective of the FIFA 11+ program is not necessarily to enhance physical performance but rather to familiarize players with proper movement techniques, methods, and body alignments to reduce the risk of injuries. In contrast, soccer practice typically involves intense physical activities that demand a high level of effort and performance from the players. As such, the FIFA 11+ program may not provide the same level of intensity and specificity required to significantly enhance athletic performance, especially in areas such as sprinting, agility, and explosive power. Therefore, it is important to recognize that the FIFA 11+ program primarily serves as a warm-up strategy. While it may have some positive effects on physical performance, its main focus is on reducing injury risk by promoting proper movement patterns and body alignments.

It is interesting to note that the sprint test results of amateur soccer players showed significant improvements only after 4 weeks of following the FIFA 11+ program, as reported by Ayala et al. (2017). This finding aligns with the outcomes of previous studies (Rössler et al., 2016; Panagoulis et al., 2020). In particular, Ayala et al. (2017) highlighted that amateur soccer players experienced notable enhancements in sprinting speed after engaging in the FIFA 11+ program for 4 weeks, with three sessions per week. This improvement in sprint performance is consistent with previous research that suggested the effectiveness of plyometric training in reducing short sprint times (typically less than 20 m), especially among athletes in the pre-peak height velocity stage (Wang and Zhang, 2016; Beato et al., 2018). Lloyd et al. (2016) previously found that plyometric training, which includes exercises such as double-leg hops in various directions and countermovement jumps for maximum height (similar to components of the FIFA 11+ program), can stimulate neural coordination and central nervous system maturation during the prepubescent stage. This neural demand and training stimulus may contribute to the observed improvements in sprinting performance among soccer players who undergo programs like the FIFA 11+ program.

The observed improvement in physical performance can be attributed to the specific characteristics of the FIFA 11+ program, which includes a range of exercises such as balancing, jumping, squatting, bounding, cutting, and muscle strength training. These exercises have the potential to improve various aspects of physical performance, including strength, neuromuscular recruitment, and muscle coordination (Chimera et al., 2004). These aspects of physical performance can play a significant role in improving leg power, speed, and agility.

The effectiveness of the FIFA 11+ program in enhancing athletic performance (particularly the 10-m sprint and the MAT) can be further understood in the context of plyometric exercises. As a rule, the more specific a plyometric exercise is to the stretch rate and load characteristics of the sport movement, the greater the transfer of the training effect to performance (Cormie et al., 2011; Sáez de Villarrea et al., 2012). Equally, in the FIFA 11+ program, there are some dynamic exercises, particularly in part 2, which may adequately optimize the neuromuscular system and improve proprioception, muscular activity, and joint stability (Hübscher et al., 2010). These underlying mechanisms are theorized to elicit specific adaptations in the neural drive, rate of neural activation, and inter-muscular control, which results in an improved rate of force development (Cormie et al., 2011). Additionally, the inclusion of sprinting, agility, and plyometric exercises alongside neuromuscular exercises in the FIFA 11+ program, which also emphasizes the importance of correct techniques in various movements (Impellizzeri et al., 2013; Akbari et al., 2019), aligns well with this principle.

The observed increase in core temperature following the FIFA 11+ warm-up, as reported by Zois et al. (2011), is indeed consistent with the improvements in various performance measures such as 20-m sprints, agility, vertical jump, and stiffness. However, we acknowledge that there are also adaptations not associated with temperature, such as post-activation potentiation (PAP), which can contribute to performance improvements. These findings are in line with similar studies (Mohr et al., 2004; Brown et al., 2008), which also indicated that warm-up routines can lead to enhanced performance. Another notable benefit of the FIFA 11+ warm-up is the increase in baseline VO_2max_, as suggested by Bizzini et al. (2013). This increase in aerobic capacity may reduce the anaerobic contribution during the initial stages of subsequent exercises. By focusing on oxidative mechanisms at the beginning of the task, there is a potential reduction in the oxygen deficit and a decrease in the contribution of anaerobic energy sources necessary for subsequent anaerobic activities. These effects can contribute to an improved endurance related to the ability to recover during and after high-intensity efforts.

While our study identified a significant improvement within the FIFA group compared to baseline measurements, it is important to acknowledge the absence of a statistically significant difference between the FIFA group and the control group. This apparent lack of difference may be attributed to several factors that warrant consideration.

• **Small sample sizes:** Both groups had relatively small sample sizes (FIFA group, *n* = 13; control group, *n* = 12). This limited sample size may have reduced the statistical power to detect differences between the groups, even if true differences exist.
• **Baseline characteristics:** The participants in both groups had similar baseline characteristics, such as age, fitness level, and physical performance. The initial similarity between the two groups may have posed a challenge in detecting significant differences resulting from the intervention.
• **Variability within groups:** Despite significant improvements within the FIFA group, there may have been substantial variability or individual responses to the intervention. This within-group variability could have obscured the between-group differences. Some participants within the control group may naturally show improvements over time, and some within the intervention group may not respond as expected. This individual variability can make it challenging to observe significant differences at the group level.
• **Duration of intervention:** The duration and intensity of the FIFA group’s intervention may not have been sufficient to generate significant differences between the groups during the study’s timeframe. Longer or more intensive interventions might be required to observe notable effects.

While our study did not reveal statistically significant differences between the FIFA and control groups, the observed significant improvement within the FIFA group indicates that further investigation is warranted. Future research should aim to investigate the effects of longer-term applications of the FIFA 11+ program on a wide range of physical performance variables and soccer-related skills. These studies should employ randomized control trial designs to ensure rigorous scientific investigation. Additionally, it would be valuable to examine the impact of the FIFA 11+ program on the performance of young athletes, specifically considering their maturity status. Young athletes may respond differently to training interventions due to their developmental stage, and understanding these effects is crucial. Furthermore, future studies should seek to uncover the mechanisms underlying the observed reduction in injury incidence associated with the FIFA 11+ program. This could involve investigating the influences of the program on neuromuscular control, biomechanics, and other factors related to injury risk in various team sports, as suggested by Gomes Neto et al. (2017). By conducting well-designed and comprehensive research in these areas, we can further validate the improvements in physical performance associated with the FIFA 11+ program and gain a deeper understanding of the contribution of the program to reducing injuries in collective sports.

### 4.1 Strengths and limitations of the study

Despite this study being the first to be realized in elite seven-a-side soccer players, it remains unclear whether the current findings can reflect general data also for elite eleven-a-side soccer players or rather represent only a team of elite seven-a-side soccer players. This is why prudence must be taken when generalizing these results. Another important limitation was the small sample size used in both the control and experimental groups. In our study, a significant number of dropouts occurred primarily due to injuries that occurred during the intervention, particularly during weekend matches and training sessions. Participants were engaged in intensive training 4–5 days a week, in addition to participating in competitive weekend matches. The nature and frequency of these activities increased the likelihood of injuries. Consequently, five participants from the FIFA group and two from the control group were injured, leading to an imbalance in group sizes. This high dropout rate affected the statistical power of our study and may impact the findings. To address this limitation, we recommend that future research should explore strategies for mitigating injuries and dropouts, including more extensive injury prevention protocols and modified interventions. These recommendations are intended to enhance the quality and practicality of future studies. However, although the sample size that was recruited for this experiment was small, significant main effects were observed. The nature of the study is based on examining these variables at a specific point in time. However, further profiling studies of elite male and female youth soccer players are necessary in order to establish reference data about the long-term FIFA 11+ program. Future research studies should take this into account and attempt to follow changes in a reproducible manner over a long-term period.

## 5 Conclusion

The experimental research presented in this study has confirmed that the 10-week FIFA 11+ program led to notable improvements in the 10-m sprinting performance and reduced the time spent to perform the MAT, particularly among elite seven-a-side soccer players, when compared to regular training. These findings align with previous research studies, indicating the FIFA 11+ program as an effective training protocol for enhancing physical performance in soccer players. However, it is worth noting that some parameters, like the 5-m sprint and FJT (between-group analysis), did not show significant improvements, highlighting the complexity of performance enhancement.

In light of these outcomes, there is a compelling case for further studies that delve deeper into the practical implementation of the FIFA 11+ program in real-world soccer settings. These investigations should address various factors, including training frequency, duration, and adherence. Such research will provide valuable insights into how to maximize the benefits of this protocol while considering the multifaceted nature of physical performance in soccer. This balanced approach, addressing both its strengths and areas of non-improvement, will guide coaches and athletes in optimizing their training regimens for comprehensive performance enhancement.

## Data Availability

The original contributions presented in the study are included in the article/Supplementary Material; further inquiries can be directed to the corresponding author.
